# Molecular and phenotypic biomarkers of aging

**DOI:** 10.12688/f1000research.10692.1

**Published:** 2017-06-09

**Authors:** Xian Xia, Weiyang Chen, Joseph McDermott, Jing-Dong Jackie Han

**Affiliations:** 1 Key Laboratory of Computational Biology, CAS Center for Excellence in Molecular Cell Science, Collaborative Innovation Center for Genetics and Developmental Biology, Chinese Academy of Sciences-Max Planck Partner Institute for Computational Biology, Shanghai Institutes for Biological Sciences, Chinese Academy of Sciences, Shanghai, China; 2 School of Information, Qilu University of Technology, Jinan, China

**Keywords:** physiological age, phenotypic, molecular, age-associated diseases, aging process

## Abstract

Individuals of the same age may not age at the same rate. Quantitative biomarkers of aging are valuable tools to measure physiological age, assess the extent of ‘healthy aging’, and potentially predict health span and life span for an individual. Given the complex nature of the aging process, the biomarkers of aging are multilayered and multifaceted. Here, we review the phenotypic and molecular biomarkers of aging. Identifying and using biomarkers of aging to improve human health, prevent age-associated diseases, and extend healthy life span are now facilitated by the fast-growing capacity of multilevel cross-sectional and longitudinal data acquisition, storage, and analysis, particularly for data related to general human populations. Combined with artificial intelligence and machine learning techniques, reliable panels of biomarkers of aging will have tremendous potential to improve human health in aging societies.

## Introduction: Why do we need biomarkers of aging?

Aging is the time-dependent physiological functional decline that affects most living organisms, which is underpinned by alterations within molecular pathways, and is also the most profound risk factor for many non-communicable diseases. To identify biomarkers of aging would, on one hand, facilitate differentiation of people who are of the same chronological age yet have variant aging rates. Quantitative biomarkers of aging could also define a panel of measurements for ‘healthy aging’ and, even further, predict life span. On the other hand, biomarkers of aging could also assist researchers to narrow their research scope to a specific biological facet in their attempts to explain the biological process behind aging or aging-related diseases. Here, we review the phenotypic and molecular biomarkers of aging. Phenotypic biomarkers can be non-invasive, panoramic, and easy to obtain, whereas molecular biomarkers can reflect some of the molecular mechanisms underlying age status. This review is centered on humans (with mouse and nematode in some rare cases).

## Molecular biomarkers of aging

This section is inspired by two high-impact reviews on the hallmarks of aging ^1, 2^. Following the framework of these reviews, we focus on developments since 2013. The American Federation for Aging Research (AFAR) has proposed the following criteria for a biomarker of aging: (1) it must predict the rate of aging; (2) it must monitor a basic process that underlies the aging process, not the effects of disease; (3) it must be able to be tested repeatedly without harming the person; and (4) it must be something that works in humans and in laboratory animals.

Biomarkers fulfilling all of the criteria proposed by the AFAR are unlikely to exist ^3^, so in the molecular part of this review we follow the first two criteria: a biomarker should predict the rate of aging, and it must monitor a basic process that underlies the aging process. For the first criterion, we required the biomarker to be correlated with aging; for the second criterion, we have organized the first part of this review according to the molecular pathways underling aging.

### DNA and chromosomes

***Telomeres.*** Telomeres are ribonucleoprotein complexes at the end of chromosomes and become shorter after each replication, as telomerase, the enzyme responsible for its replication, is not regularly expressed in somatic cells ^4^. The length of telomeres in leukocytes has been associated with aging and life span ^5^ as well as age-related diseases, such as cardiovascular diseases ^6, 7^, cancer ^8^, and neurological disorders ^9^.

***DNA repair.*** The link between DNA damage and repair has been implicated in aging by the accumulation of senescent cells ^10^ or genomic rearrangements ^11^. More recently, this link was directly demonstrated, and controlled induction of DNA double-strand breaks in mouse liver inducing aging pathologies and gene expression was shown ^12^. Immunohistochemistry of γ-H2A.X is an established quantitative biomarker of aging because H2A.X is a variant of the H2A protein family, and phosphorylated H2A.X, γ-H2A.X, is an initial and essential component of DNA damage foci and therefore a reliable marker of the extent of DNA damage ^13– 15^. Serum markers of DNA damage, including CRAMP, EF-1a, stathmin, N-acetyl-glucosaminidase, and chitinase, have also been established ^16^. Of note, the dermal fibroblasts from centenarian donors were shown to be less sensitive to H _2_O _2_-induced DNA damage than fibroblasts from young and old donors ^17^. Such *ex vivo* experiments could also be a potential biomarker of aging.

***Epigenetic modifications.*** Age-related changes in DNA methylation patterns, notably as measured by the epigenetic clock, are among the best-studied aging biomarkers ^18– 20^. Analysis of methylation profiles in the blood found that only three CpG sites could predict age with a mean absolute deviation from chronological age of less than 5 years ^21^. The association between age and DNA methylation can be extended to age-associated diseases, such as diabetes ^22^. For a full review of the epigenetic regulation of aging, see Sen *et al*. ^23^.

### RNA and transcriptome

***Transcriptome profiles.*** With rapid progress in single-cell RNA sequencing (RNA-seq) technology, it has begun to be applied to the study of biomarkers of aging. Lu *et al*. have recently shown that cell-to-cell expression variation, as measured by single-cell RNA-seq of high-dimensional flow cytometry sorted T cells, is associated with aging and disease susceptibility ^24^.

A recent study used whole-blood gene expression profiles from 14,983 individuals to identify 1,497 genes with age-dependent differential expression and then used them to calculate the ‘transcriptomic age’ of an individual, suggesting that transcriptome signatures can be used to measure aging ^25^.

***Non-coding RNAs.*** MicroRNAs (miRNAs) are a class of small (21- to 23-nucleotide) non-coding RNAs that, through base-pairing mechanisms, regulate a broad range of biological processes, including metabolism ^26^ and aging ^27^. Among them, circulating miRNAs can be stable in plasma by residing in exosomes or binding to protein or lipoprotein factors, thus making them easy-to-access biomarkers. miR-34a was the first observed circulating miRNA with an altered expression pattern during mouse aging ^28^. Its expression is found to correlate with age-related hearing loss in mice and humans ^29^. miR-21 was defined as an inflammatory biomarker in a study of 365 miRNAs in the plasma of healthy and old humans ^30^. miR-151a-3p, miR-181a-5p, and miR-1248 are reported to be significantly decreased with age in humans, in which all three miRNAs also show indications of associations with inflammation ^31^. miR-126-3p has been found to be positively correlated with age in 136 healthy subjects from 20 to 90 years of age ^32^. Through expression of GFP driven by miRNA promoters, Pincus *et al*. found that levels of *mir-71*, *mir-246*, and *mir-239* in early adulthood vary across individuals and are predictive of life span ^33^. A recent review ^27^ summarized the associations of other types of circulating non-coding small RNAs, such as tRNA and YRNA.

Long non-coding RNAs (lncRNAs) are a heterogeneous class of non-coding RNAs which are defined as transcripts longer than 200 nucleotides and devoid of evident open reading frames ^34^. Two recent reviews summarize the role of lncRNAs in aging ^35, 36^. The diverse functional mechanisms of lncRNA are beyond the scope of this review, and readers may consult a recent review on this topic ^37^; here, we list lncRNAs that function in aging. The lncRNA MIR31HG was identified to be upregulated in oncogene-induced senescence and required for polycomb group–mediated repression of the INK4A locus ^38^. Downregulation of lncRNA AK156230 occurs in replicative senescence and its knockdown in mouse embryonic fibroblasts induces senescence through dysregulation of autophagy and cell cycle pathways, as shown by expression profiles ^39^. Meg3 is upregulated during cardiovascular aging as well as in senescent human umbilical venous endothelial cells ^40^. As most of the lncRNAs studies have been anecdotal, high-throughput lncRNA studies, such as CRISPR-Cas9 screen of functional lncRNAs ^41^, will be a useful future step toward understanding lncRNA functions in the aging process.

### Metabolism

That dietary restriction is the most conserved means to extend life span and health span from yeast to mammals ^42^ points to a pivotal role of metabolism in aging regulation and to the potential for metabolic factors to be biomarkers.

***Nutrient sensing.*** The insulin/insulin-like growth factor 1 (IGF-1) signaling (IIS) pathway, which participates in glucose sensing, is the earliest discovered and the most well-known pathway to antagonize longevity. Paradoxically, IGF-1 declines in wild-type mice or mouse models of premature aging whereas attenuating IIS activity extends life span ^43^. Such observations led to the potential inclusion of IIS pathway members, such as growth hormone and IGF-1, as biomarkers of aging ^44, 45^.

The mechanistic target of rapamycin (mTOR) protein senses high amino acid concentrations. Inhibition of mTOR can extend life span ^46^. Unlike the IIS pathway, mTOR activity increases with age in the ovarian surface epithelium of aged human and mouse ovaries, which contributes to pathological changes ^47^. Phosphorylated S6 ribosomal protein (p-S6RP, or pS6) is a downstream target and also a known marker of active mTOR signaling ^47, 48^, which is a potential biomarker of aging as indicated in the research of aged ovaries ^47^.

In contrast to IIS and mTOR function, 5′-adenosine monophosphate (AMP)–activated protein kinase (AMPK) and sirtuins sense nutrient scarcity instead of abundance. AMPK detects high AMP levels whereas sirtuins are sensors of high NAD ^+^ levels, and both mark low-energy states. The upregulation of AMPK activity by metformin, a drug for type II diabetes, could mimic some of the benefits of caloric restriction, and metformin extends life span in male mice ^49^. AMPK is upregulated with age in skeletal muscles ^50^.

Sirtuins have the ability to directly link cellular metabolic signaling (reflected by NAD ^+^) to protein post-translational modifications through a chemical reaction (deacetylation of lysine). During aging, NAD ^+^ is reduced ^51^ and sirtuins are downregulated ^52, 53^. An analysis of primary human dermal fibroblasts found that SIRT1 and SIRT6 are downregulated through passaging ^54^. Similarly, levels of SIRT1, SIRT3, and SIRT6 detected by Western blotting showed significant decrease in ovaries of aged mice ^55^. In human peripheral blood mononuclear cells, SIRT2 also decreases with age ^56^.

***Protein metabolism.*** Protein carbamylation is one of the non-enzymatic post-translational modifications which occur throughout the whole life span of an organism, leading to tissue accumulation of carbamylated proteins ^57^. It is considered a hallmark of molecular aging and is related to aging-related diseases, such as cardiovascular disease ^58^.

Advanced glycation end products (AGEs) are a heterogeneous group of bioactive molecules that are formed by non-enzymatic glycation of proteins, lipids, and nucleic acids ^59^. Accumulation of AGEs in aging tissues leads to inflammation ^60^, apoptosis ^61^, obesity ^62^, and other age-related disorders ^63^. AGEs can be detected via high-performance liquid chromatography, gas chromatography-mass spectrometry, and immunochemical techniques ^64^. N-glycans are a class of glycoproteins with sugar chains bonded to the amide nitrogen of asparagine. The spectrum of N-linked glycans (the N-glycome) can now be investigated because of the development of high-throughput methods. The accumulation of N-linked glycation at Asn297 of the Fc portion of IgG (IgG-G0) can contribute to low-grade pro-inflammatory status in aging ^65^.

***Lipid metabolism.*** Triglycerides are found to increase monotonously with age and thus could be a biomarker of aging ^66^. Studies of serum samples by shotgun lipidomics found that phospho/sphingolipids are putative markers, and biological modulators, of healthy aging ^67^. However, the design of these studies is questionable in that they have a group of elderly individuals as a ‘not healthy aging control’ and compare them with the ‘successful aging’ centenarian group ^67, 68^, but the two groups are obviously of very different ages. Therefore, it is not clear whether it was the age difference or the success of healthy aging that contributed to the differences in lipidomics.

### Oxidative stress and mitochondria

Biomarkers of oxidative stress have long been regarded as a class of aging biomarkers. The products of oxidative damage to proteins include o-tyrosine, 3-chlorotyrosine, and 3-nitrotyrosine. 8-iso prostaglandin F _2α_ is a biomarker for phospholipid damage. 8-hydroxy-2′-deoxyguanosine and 8-hydroxyguanosine are produced by the oxidative damage of nucleic acids ^69^. The concentration of these biomarkers in body fluids can be detected via high-performance liquid chromatography and mass spectrometry. Shen *et al*. engineered a circularly permuted yellow fluorescent protein (cpYFP) expressed in *Caenorhabditis elegans* mitochondrial matrix as a sensor of oxidative stress and metabolic changes; the authors found that adult day 3 mitochondrial cpYFP flash frequency is a good predictor of *C. elegans* life span under different genetic, environmental, and stochastic conditions ^70^.

Although free radicals, the source of oxidative stress, are mainly produced in mitochondria, dysfunctional mitochondria can contribute to aging independently of reactive oxygen species. To measure mitochondria function, blood- and-muscle based respirometric profiling strategies are available, and the association of this potential reporter with bioenergetic capacity of other tissues ^71^ or phenotypes, such as gait speed ^72^, has been investigated. Extracellular mitochondria components can function as damage-associated molecular pattern molecules (DAMPs) (see also “Inflammation and intercellular communication”) and these induce neuroinflammation when injected in mouse hippocampus ^73^.

### Cell senescence

In mitotic tissues, the gradual accumulation of senescent cells is thought be one of the causal factors of aging ^74– 76^. Thus, the biomarkers of cell senescence can also be used as markers. Such biomarkers have been summarized in recent reviews ^77, 78^. The most widely used marker is senescence-associated β-galactosidase (SAβ-gal) ^79^ and p16 ^INK4A^ ^80, 81^. SAβ-gal reflects increased lysosomal mass ^82^ but can yield false positives because of its low specificity ^83^. SAβ-gal is a cell damage marker, and p16 ^INK4A^ is required to induce, and is indicative of, permanent cell cycle arrest ^81^.

Other senescent cell markers include activated and persistent DNA-damage response (see “DNA repair”), telomere shortening and dysfunction (see “Telomere”), and senescence-associated secretory phenotype (SASP) (see “Inflammation and intercellular communication”).

### Inflammation and intercellular communication

SASP is a consequence of cell senescence and may occur in cells that, though undergoing cell cycle arrest, are still metabolically active and secrete proteins. SASP functions in an autocrine/paracrine manner ^84, 85^. The major components of SASP factors are soluble signaling factors, including interleukins, chemokines, and growth factors. Proteins that are associated with the SASP, such as interleukin-6, tumor necrosis factor-alpha, monocyte chemoattractant protein-1, matrix metalloproteinases, and IGF binding proteins, increase in multiple tissues with chronological aging and occur in conjunction with sterile inflammation ^86^. Comprehensive catalogs of SASP also include secreted proteases and secreted insoluble proteins/extracellular matrix components and are summarized by Coppé *et al*. ^87^ and the Reactome database ( http://www.reactome.org/content/detail/R-HSA-2559582).

The DAMPs, such as heat shock proteins, histones, high-mobility group box 1, and S100, compose a class of molecules released after injury or cellular death ^88^ and mediate immune response. The association between DAMPs and other hallmarks of aging has been reviewed by Huang *et al*. ^89^.

## Phenotypic biomarkers of aging

Still following the criteria proposed by the AFAR ^3^, here we categorize the phenotypic biomarkers of aging. It is difficult for phenotypic biomarkers to monitor a basic molecular process that underlies the aging process, so we follow three standards: a biomarker should predict rate of aging, it must be able to be tested repeatedly without harming the person, and it monitors one or more physiological processes.

Physical function and anthropometry are the most practical measurements among phenotypic biomarkers of aging. In this regard, walking speed, chair stand, standing balance, grip strength, body mass index, waist circumference, and muscle mass are well known ^90^. These physical functional measurements, though simple, can actually perform better than DNA methylation in terms of relationship to health status in demographic research ^91^.

Quantitative phenotypes of external human features also show significant relationships with aging ^92, 93^. Quantified facial features based on three-dimensional (3D) facial images, such as mouth width, nose width, and eye corner droop, are highly associated with age. In fact, 3D facial images can be used to quantify the biological age of an individual ^92^.

### Integration of aging biomarkers

Biomarkers of aging can be used to predict the physiological age, which reflects their state of health, via statistics and machine learning algorithms. A single class of biomarkers, which is intrinsically a matrix of features, can be used in the prediction. DNA methylation was used to predict age with an error of about 3.6 years using 8,000 samples ^94^. 3D facial images have also been used to predict age with a mean deviation of 6 years ^92^.

Integration of multiple biomarkers can be even more powerful. The Dunedin Study ^91^ has focused on middle-aged people and used different measurements (telomere lengths, epigenetic clocks, and clinical biomarker composites) and compared their performance in predicting health status, as measured by physical functionality, cognitive decline, and subjective signs of aging. The three types of measurements in this study do not correlate with each other, suggesting that there is no single index of biological age. Therefore, another approach is to use statistic distance, *D _M_* ^95, 96^, to assess the degree of deviation of an individual’s biomarker profile from the reference population. *D _M_* is the Mahalanobis distance ^97^ of multi-variants (in the simplest case, when all the variants are uncorrelated, this distance is the sum of the absolute values of z-scores), and is proven to be insensitive to biomarker choice across 44 available markers and to be generalizable with multiple marker variants. Recently, a modular ensemble of 21 deep neural networks was used to predict age by using measurements from basic blood tests by training over 60,000 samples, which revealed the five most important blood markers for predicting human chronological age: albumin, glucose, alkaline phosphatase, urea, and erythrocytes ^98^.

## Conclusion and outlook

As expected from the complex nature of the aging process, aging biomarkers are multilayered and multifaceted and consist of a dizzying array of parameters, which we further summarized in an even more concise form as a table ( Table 1). This, however, does not mean that they are equally useful. We need to point out that not all factors, although they might be involved in the underling biological process of aging, are proven to be useful in terms of measuring human aging at this point.

**Table 1.** Biomarkers of aging. For species source, if there is one in humans, then other model organisms are omitted.

	Biomarker Category	Biomarker Subcategory	Biomarker	Trend with age	Species
**Molecular biomarkers**	DNA and chromosome	Telomere	Leukocyte telomere length	Decrease	Human
		DNA repair	γ-H2A.X immunohistochemistry	Increase	Human
		Epigenetic modification	DNA methylation	Global hypomethylation and local hypermethylation	Human
	RNA and transcriptome	Transcriptome profiles	Heterogeneity of CD38 in CD4 ^+^CD27 ^+^ T cells	Decrease	Human
			Heterogeneity of CD197 in CD4 ^+^CD25 ^+^ T cells	Increase	Human
		Circulating microRNAs (miRNAs)	miR-34a, miR-21, miR-126-3p	Increase	Human
			miR-151a-3p, miR-181a-5p, miR-1248	Decrease	Human
		Long non-coding RNAs	MIR31HG	Increase in cell senescence	Human
			AK156230	Decrease in cell senescence	Mouse
			Meg3	Increase in cell senescence	Human
	Metabolism	Nutrient sensing	Growth hormone and insulin/insulin- like growth factor 1 (IGF-1)	Decrease	Human
			Mechanistic target of rapamycin (mTOR) and pS6RP	Increase	Human
			NAD ^+^, SIRT1, SIRT2, SIRT3, SIRT6	Decrease	Human
		Protein metabolism	Protein carbamylation, such as homocitrulline rate	Increase	Human
			Advanced glycation end products and N-glycans	Increase	Human
		Lipid metabolism	Triglycerides	Increase	Human
	Oxidative stress and mitochondria		o-tyrosine, 3-chlorotyrosine, 3-nitrotyrosine, 8-iso prostaglandin F2α, 8-hydroxy-2′-deoxyguanosine, 8-hydroxyguanosine	Increase	Human
	Cell senescence		Senescence-associated β-galactosidase	Increase in cell senescence	Human
			p16INK4A	Increase in cell senescence	Human
	Inflammation and intercellular communication		Senescence-associated secretory phenotype	Increase	Human
**Phenotypic** **biomarkers**		Physical function and anthropometry	Walking speed, chair stand, standing balance, grip strength, muscle mass	Decrease	
			Body mass index, waist circumference	Increase	
		Facial features	Mouth width	Increase	
			Nose width	Increase	
			Mouth-nose distance	Increase	
			Eye corner slope	Decrease	

Recently, the MARK-AGE project was announced as a large-scale integrated project aimed to find a powerful set of biomarkers for human aging based on over 3,200 subjects ^99^. Although more details from this project remain to be seen, the pace of identifying and using biomarkers of aging to improve human health, preventing aging-associated diseases, and extending healthy life span will only be further increased by the myriad of data generated. These include not only data from large human cohort studies but also ordinary people’s genomic, functional genomic, phenotypic, and lifestyle data, which will be facilitated by the ever-growing capacity of data acquisition, storage, and analysis. It would not be far-fetched for there one day to be an artificial intelligence program capable of precise prognosis of how long a person can live, based on his or her quantitative measurements in a large panel of biomarkers of aging.
